# Minimizing Deformations during HP MJF 3D Printing

**DOI:** 10.3390/ma16237389

**Published:** 2023-11-28

**Authors:** Karel Ráž, Zdeněk Chval, Sacha Thomann

**Affiliations:** 1 Faculty of Mechanical Engineering, Regional Technological Institute, University of West Bohemia, Univerzitni 8, 306 14 Plzen, Czech Republic; zdchval@fst.zcu.cz; 2 Polytech Montpellier, MI4, Université de Montpellier, Pl. Eugène Bataillon, 34090 Montpellier, France; sacha.thomann@etu.umontpellier.fr

**Keywords:** 3D printing, additive manufacturing, PA12GB, Digimat, deformation, MJF

## Abstract

(1) Background: The purpose of this study was to investigate deformations that occur during additive manufacturing by the HP (Hewlett-Packard) Multi Jet Fusion (MJF) process. These deformations affect the final properties of 3D-printed parts, and proper compensating technology has to be developed in order to minimize these deformations. (2) Methods: Parts were printed with powder composed of nylon plastic infused with glass beads (PA12GB). The HP MJF technology was used during investigations. All parts (specimens) were measured at different points over an extended period to follow the deformations at each point. Different finite element simulations were performed to compare them with real results and assess the viability of using simulations to save time. Various modules of the Digimat software, such as additive manufacturing (AM), material focused (MF), finite element (FE), and computer-aided engineering (CAE), were used to run the simulations. (3) Results: It was found that the printing position of the part in the printer had an impact on deformations. When the part was simulated in a tilted position but alone (deformation: 7.19 mm), the value of the deformation was 1.49 mm greater than when the other parts (two comparable parts) were simulated at the same time (deformation: 5.7 mm). The difference between the simulation with the three parts together (deformation: 5.7 mm) and reality (deformation: 3.44 mm) was 2.26 mm. Finally, the difference between the simulated single part (deformation: 7.19 mm) and the real part (deformation: 3.44) was 3.75 mm. (4) Conclusions: The results of this study will contribute to a better understanding of deformation mechanisms and will suggest solutions for improving the quality of printed parts. Three-dimensional printing is a rapidly growing technology that offers numerous possibilities across various fields. However, one commonly encountered issue is the deformation of printed parts. Methods for minimizing deformations were studied during the 3D printing process using HP MJF technology. Various factors contributing to deformation were investigated, and different techniques for reducing them were explored.

## 1. Introduction

Three-dimensional printing of industrial parts is more and more used to create complex and cheaper products. Additive manufacturing has advantages like design freedom and cost reduction, but there are also disadvantages like the printing time and the appearance of warping deformation in the part due to high temperature [1,2]. There exist different types of additive manufacturing technologies, such as SLS, FDM, FFF, or MJF. For each of these printing techniques, there are different deformations after the printing process [3,4]. This research is focused on deformation and warpage prediction using FEM simulation tools. Deformation after the production process can occur due to various effects (high temperature, wrong positioning in printing area, etc.).

Deformation in 3D-printed parts is a common issue and can occur for various reasons.

Understanding this mechanism of deformation is crucial for improving the quality and reliability of 3D-printed components. Common problems associated with deformation in 3D-printed parts are warping (caused mainly by different cooling rates across the part), cracking (caused by internal stresses), shrinkage (caused by thermal contractions), and layer misalignments. This research is focused on shrinkage and warping [5].

Additive manufacturing (AM) has revolutionized the way we design, produce, and interact with objects, opening up new horizons in various industries. One of the most promising techniques in the field of AM is the high-performance Multi Jet Fusion (HP MJF) process [6,7], which has gained significant attention for its ability to fabricate complex, functional 3D parts with exceptional speed and precision. However, like any manufacturing process, HP MJF is not without its challenges and limits. One critical aspect that demands a comprehensive understanding is the potential deformation of 3D parts during the printing process.

**Figure 1:**
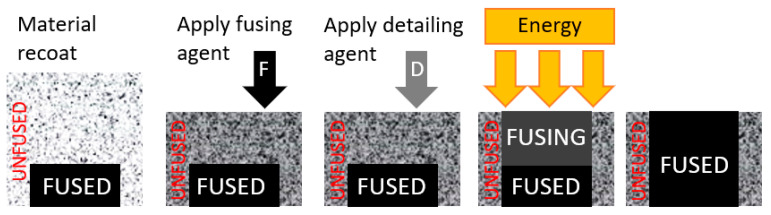
A diagram of the MJF process.

In this article, the deformation in 3D parts produced through the HP MJF process (see Figure 1) is deeply studied and described. The underlying mechanisms that lead to distortion and warping, such as residual stresses, are explored. Understanding these factors is crucial for optimizing printing parameters and ensuring the manufacturability of complex geometries [8,9,10].

The influence of the production method on part deformation and strength is investigated so that engineers can leverage this knowledge to design parts that are more resilient to deformation while maintaining their intended functionalities. The interplay between part orientation, support structures, and build layout plays a pivotal role in mitigating deformation and achieving successful outcomes.

In conclusion, this article aims to shed light on the phenomenon of deformation in 3D parts fabricated using the HP MJF process. By comprehending the underlying mechanisms, designers, engineers, and manufacturers can make informed decisions to minimize deformation and optimize the overall manufacturing process [11,12,13]. Through this exploration, this research aspires to unlock the full potential of the HP MJF technology in diverse industries worldwide, where complex, high-performance 3D polymer parts are at the forefront of innovation.

## 2. Materials and Methods

The HP Multi Jet Fusion (MJF) process involves powdered materials being selectively fused layer by layer to build complex 3D structures (Figure 1) [14]. This advanced 3D printing technology uses a powder-based additive manufacturing process [15,16]. A layer of powdered unfused material is spread over the built platform, and inkjet arrays selectively apply a fusing agent and detailing agent onto the powder according to the digital design. A heating element then fuses the treated areas together to form a solid layer. This layer-by-layer process continues until the 3D print is complete [17]. The final properties of the part are highly dependent on the layer quality and thickness [18,19,20].

The material considered in this research was PA12GB. The thickness of one layer of PA12GB is around 0.08 mm, and this technology results in actual productivity of 4115 cm^3^/h [21,22]. The processing temperature for PA12GB is around 160 to 180 °C [23]. This temperature has to be understood with respect to the powder melting point of PA12GB, which is 186 °C. The material PA12GB has a tensile strength of 30 MPa (according to the official datasheet). The guaranteed value of elongation at break, as provided by the producer of the material, is 6.5% [24,25].

The HP MJF technique offers impressive benefits, such as high speed, precision, and the ability to produce complex parts with excellent detail resolution and mechanical properties. It also introduces complexities related to thermal, mechanical, and material properties. These factors can contribute to varying degrees of deformation, impacting the final accuracy, dimensional stability, and overall quality of the printed parts.

HP MJF has applications in various industries, including aerospace, automotive, healthcare, and consumer goods [26]. Its versatility and efficiency make it a preferred choice for rapid prototyping, customized manufacturing, and production of end-use parts with high reliability. However, careful consideration and optimization of the printing parameters are necessary to mitigate potential deformations and ensure optimal results in the final printed parts [27].

Furthermore, the impact of the material properties on deformation behavior was examined, recognizing that different powder materials react differently under the heat and pressure of the MJF process. Identifying the ideal material and manufacturing settings and considering correct mechanical characteristics is vital for obtaining highly accurate and dimensionally stable parts [28,29].

## 3. Microstructure

To carry out this study, the digital definition of the characteristics of the used material had to be the first step. This approach would enable us to obtain a virtual representation that is as faithful as possible to reality, allowing us to anticipate the behavior expected under real conditions.

### 3.1. Material Behavior

To begin with, FEM analysis was used to numerically define the material, i.e., PA12GB. This is a composite material made by incorporating glass beads into polyamide 12 (PA12), which is a type of nylon, to reinforce the material. Material properties for the combination of PA12 and glass beads were obtained using Digimat MF (see Table 1) and the material datasheet (see Table 2).

The constitutive law used to model PA12 utilized elastoplastic law for the J2 plasticity model and exponential and linear laws for the isotropic hardening model [30]. An isotropic elastic model was used to model the glass beads [31].

Now that the material properties had been defined, the microstructure properties had to be described. The PA12GB microstructure is divided into two phases:

- Phase one: PA12 phase, which is a matrix phase;
- Phase two: Glass beads phase, which is an inclusion phase with many spherical inclusions, the total mass fraction of which is 0.4 of phase one.

The elastic domain simulation with Hooke’s law is possible according to the following equation:

*E* = *σ* × *ϵ* (1)

A failure model had to be defined within Digimat in order to generate plastic domain behavior and run the analysis. The 3D transversely isotropic Tsai–Hill model was chosen because it incorporates interactions between stress components, even though it does not take into account the tension–compression asymmetry [32,33,34], which is negligible in our case. The 3D transversely isotropic Tsai–Hill model under deformation is written in Digimat as follows [35]:

(2) $$f_{A}=\sqrt{F_{A}(\epsilon )},$$

With

(3) $$F_{A}\left(\epsilon \right)=\frac{{\epsilon^{2}}_{11}}{X^{2}}-\frac{\epsilon_{11}\left(\epsilon_{22}+\epsilon_{33}\right)}{X^{2}}+\frac{\left({\epsilon^{2}}_{22}+{\epsilon^{2}}_{33}\right)}{Y^{2}}+\left(\frac{1}{X^{2}}-\frac{2}{Y^{2}}\right)\epsilon_{22}\epsilon_{33}\;+\frac{\left({\left(2\epsilon_{12}\right)}^{2}+{\left(2\epsilon_{13}\right)}^{2}\right)}{S^{2}}+(\frac{1}{Y^{2}}-\frac{1}{4X^{2}}){\left(2\epsilon_{23}\right)}^{2}$$

where

- ϵ_11_: Principal strain in the fiber direction.
- ϵ_22_: Principal strain perpendicular to the fiber.
- ϵ_33_: Principal strain perpendicular to the fiber.
- ϵ_23_: Strain perpendicular to the fiber.
- f_A_: Failure indicator; failure occurs when indicator is more or equal to 1.
- X, Y, S are unitless maximum strain parameters.
- In our case of rounded glass beads, this equation can be simplified because the fiber is globular and its properties are the same in all three directions. This complex equation is used because of its possible use for other (nonglobular) phases [36,37].

When everything is defined, Digimat can be used to obtain the stress versus strain curve, but this is only a simulated result. Tensile tests on test specimens with various orientations during printing were performed in order to determine the material’s experimental curves. This made it possible to compare the experimental and simulated results.

**Figure 2:**
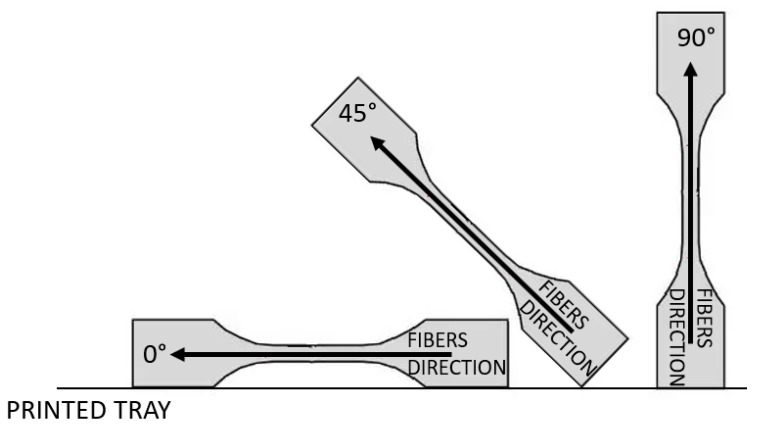
Different orientations for 3D printing.

The highest difference was for orientation 0° and 90° [38,39], while orientation 45° had an average value between the two. The example of specimen orientation is shown in Figure 2.

**Figure 3:**
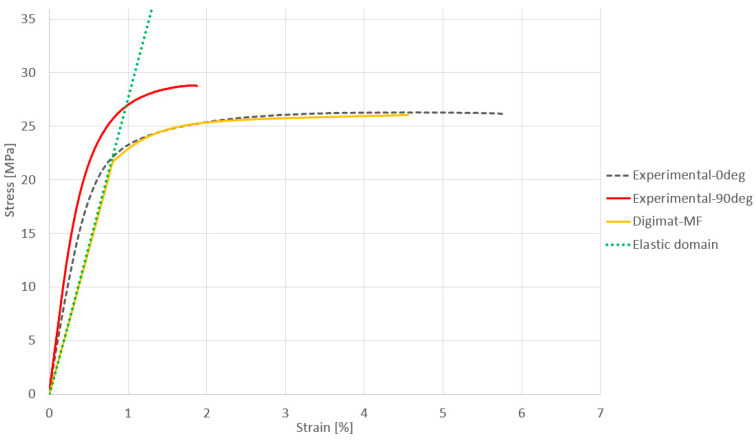
Stress–strain curves for PA12GB.

Each result is plotted onto the same graph in Figure 3. The gray dashed curve represents the stress–strain curve of PA12GB at an angle of 0 degrees, while the red curve represents the curve for PA12GB at an angle of 90 degrees. The difference was observed in the plastic domain, where failure occurred much earlier for the 90° test compared to the 0° test.

The Young’s modulus was higher for the 90° orientation, and the rupture occurred at lower strain (compared to 0°) because loading is affected more by the cohesivity between layers [40]. The linear behavior (Young´s modulus from the datasheet) of the material is shown in Figure 3 using the green dotted line. The last curve in yellow represents the curve generated by Digimat, which closely matches the tensile curve at 0°. The material created via Digimat therefore seems consistent with reality and can be used to predict deformation. The obtained values have to be comparable to the official value of tensile strength from the HP datasheet, which is 30 MPa. The difference is caused by the not absolutely homogenous printing process in reality compared to the ideal printing process achieved when obtaining the value for the datasheet. The influence of other parts within the printing area on the strength (heat transfer and temperature distribution) is significant (tensile strength decreasing by 10–15%) [41,42].

The orientation of parts during the printing process also affects the accuracy of the print. This geometric accuracy is negligible with respect to the thermal deformation caused by the design and thermal expansion of the material.

### 3.2. Microstructure Simulation

**Figure 4:**
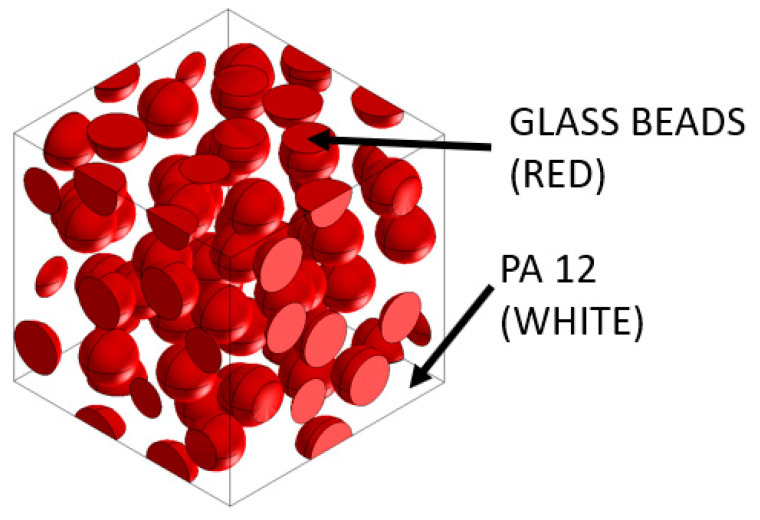
Representative volume element (RVE).

An examination of the material’s microstructure, represented by the representative volume element (as depicted in Figure 4), was conducted in order to simulate part deflection and behavior during the additive manufacturing process. This analysis aimed to visualize stress distribution within the material, thereby enhancing our internal comprehension of its properties. The stiffness and thermal properties of PA12GB results from this material modeling. These crucial values were internally used within Digimat for further simulations of the additive manufacturing process. This step was necessary for correct material modeling within FEM analysis.

**Figure 5:**
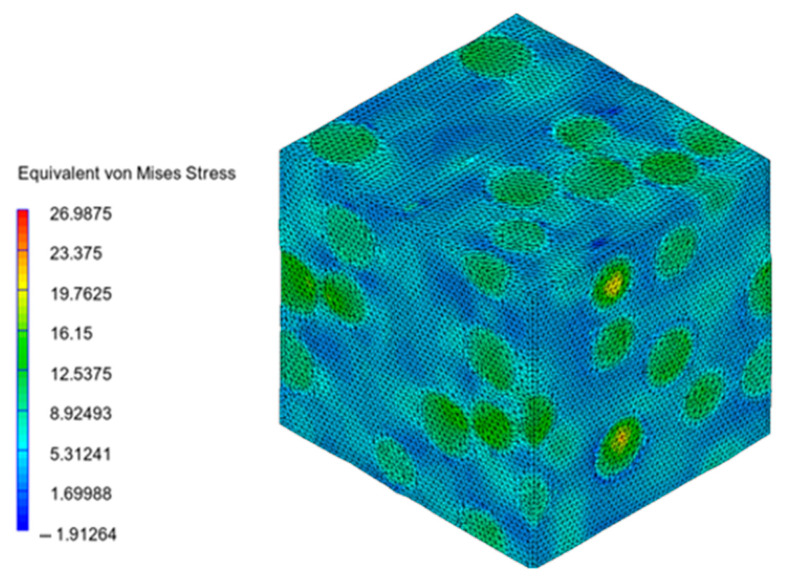
von Mises stress, σ_Vm_, in microstructure (MPa).

**Figure 6:**
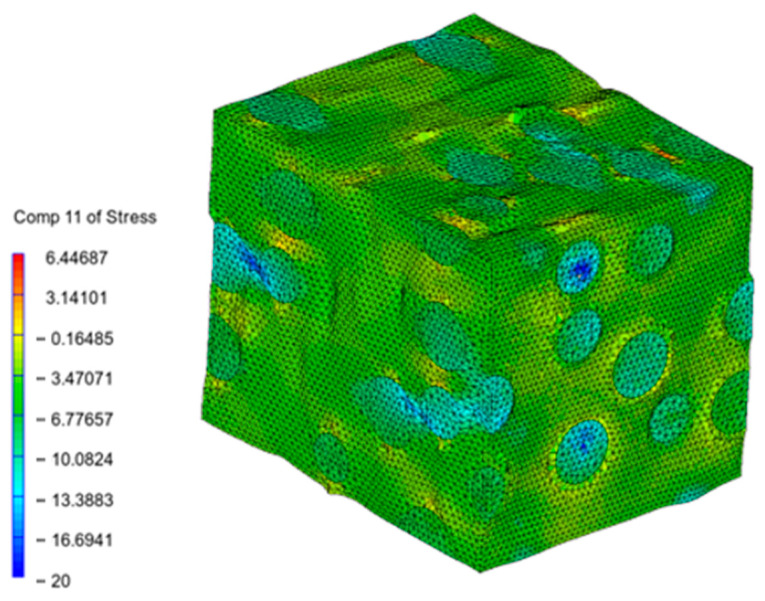
Stress in direction 11 (main direction) σ_11_ (deformation amplified 14 times) (MPa).

Digimat FE was used to generate the microstructure composed of the PA12 matrix and numerous spherical inclusions of glass beads. When a mechanical strain loading of 0.15 mm/mm was applied, von Mises stresses were obtained, as shown in Figure 5. The stress/contraction in the main direction are shown in Figure 6.

As can be seen in Figure 5 and Figure 6, the stresses were mainly distributed in the glass beads, while they were lower in the PA12 matrix.

## 4. Deflection Simulation

Simulations of 3D-printed polymer parts were carried out in order to observe the different deformations that can occur in these parts as a function of different parameters. The software Digimat 2023.3 AM was used for this task.

The first step of simulation is definition of the material characterization, followed by importation to a CAD model to properly define orientation and positioning. The simulation of the building process depends on the quality of the mesh (number of voxels). This step results in coupled thermal–structural FEM simulation, which can be used to obtain results of deflection and prediction of defects. Elements of each layer are turned on with respect to the movement of the printing head.

### 4.1. Part Design

**Figure 7:**
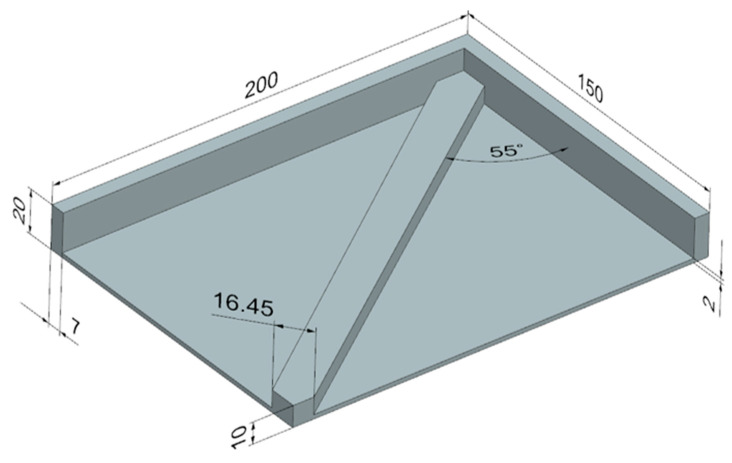
Design of the test part.

A rectangular plate with a thick edge on half the perimeter and a rib running diagonally across the piece was chosen. The part was designed in Ansys and measured 150 mm × 200 mm × 20 mm (Figure 7). It is obvious that the design of this part will lead to deformation after the printing process. This deformation is caused by the CAD design (one-sided rib). The thermal behavior of the polymer PA12GB during cooling causes shrinkage. This shrinkage is significant in areas where more materials are concentrated. Therefore, this will be the part that deforms upwards in the direction of the rib location. The correct design should be with ribs on both sides, but this design was not possible to use because of the function of the part.

### 4.2. Digimat AM Setup

To effectively simulate the deformation of the component, it is imperative to select Digimat AM parameters that correspond with real-world conditions. The printing chamber’s dimensions aligned with those employed in the HP printer (380 × 284 × 380 mm). The simulation encompassed a comprehensive full-build simulation, accounting not only for the part itself but also for the surrounding powder material [43].

As outlined in the Materials and Methods selection, we opted for PA12GB based on its technical characteristics outlined in the previous section, which closely approximate the actual material. We maintained the printing process parameters without alteration with a consistent printing temperature of 330 degrees Celsius.

**Figure 8:**
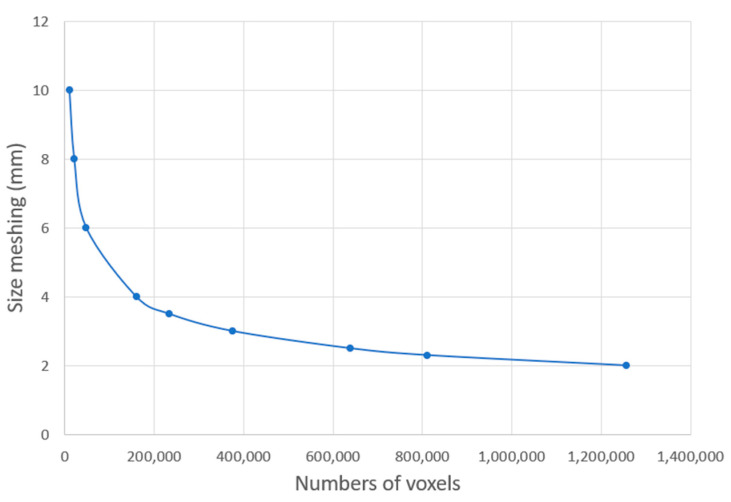
Quantity of voxels depending on mesh size.

**Figure 9:**
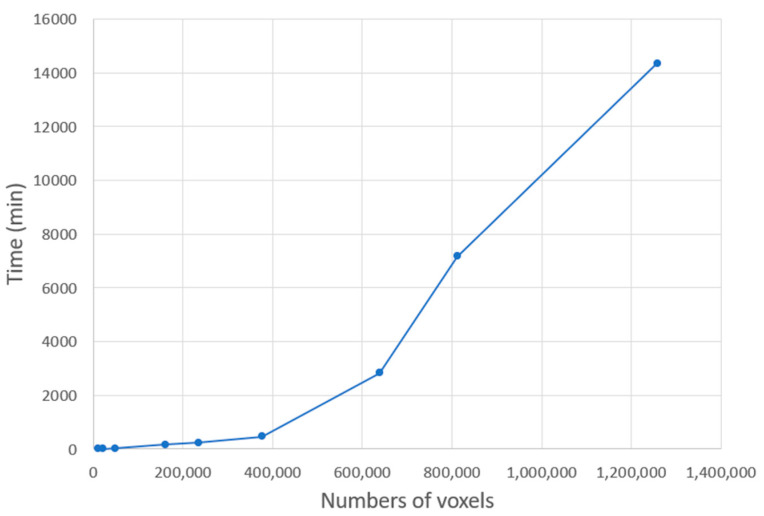
Simulation time depending on voxel number.

However, one parameter was modified. The thickness of each layer of powder, normally 0.08 mm, was increased to 1 mm for reasons relating to computer processing power and storage. For this reason, at the Digimat meshing stage of the part, the voxel size was set to 2 mm, generating a mesh with 410,688 voxels in full build. The process took 963 min, or 16 h 3 min, on a computer with an Intel(R) Core(TM) i5-4590 CPU @3.30 GHz. Given that the number of voxels increases quadratically with decreasing voxel size, this was the best compromise between time and accuracy, as shown in Figure 8 and Figure 9.

**Figure 10:**
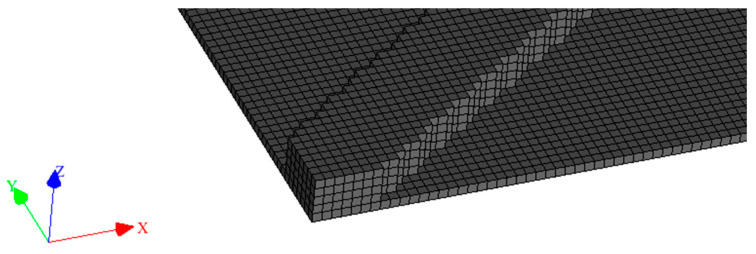
Part meshed with Digimat—detail of a corner.

Meshing was then carried out on Digimat layer by layer. The mesh quality is shown in Figure 10.

### 4.3. Results from Digimat FEM Analysis

Once the part had been meshed, three simulations were carried out to determine the differences generated by the orientation of the print. The first one was a simulation of the single part oriented horizontally (flat position) in the printing chamber. The simulation considered the coupled structural–thermal solution with element birth function. Heat transfer coefficients were set up by the solution process with respect to the selected material [44,45].

**Figure 11:**
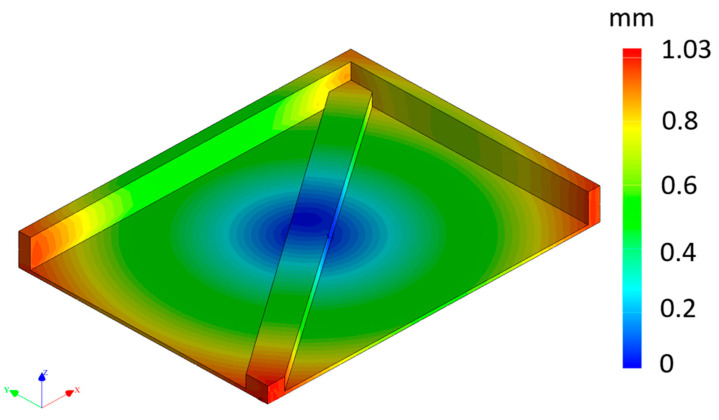
Simulated part in flat position in the printer with PA12GB (mm).

The deformations for the flat orientation occurred in a circular pattern originating from the center of the component, as illustrated in Figure 11. This occurrence was due to the cooling process, where the part initially cooled from its outer edges towards the center, causing it to contract inward to its midpoint. It is important to note that the most significant deformations occurred at the corners, where there was a lower amount of material. Nevertheless, it is worth noting that the overall deformation of the part appeared to be minimal, with a maximum deflection of only 1.03 mm.

**Figure 12:**
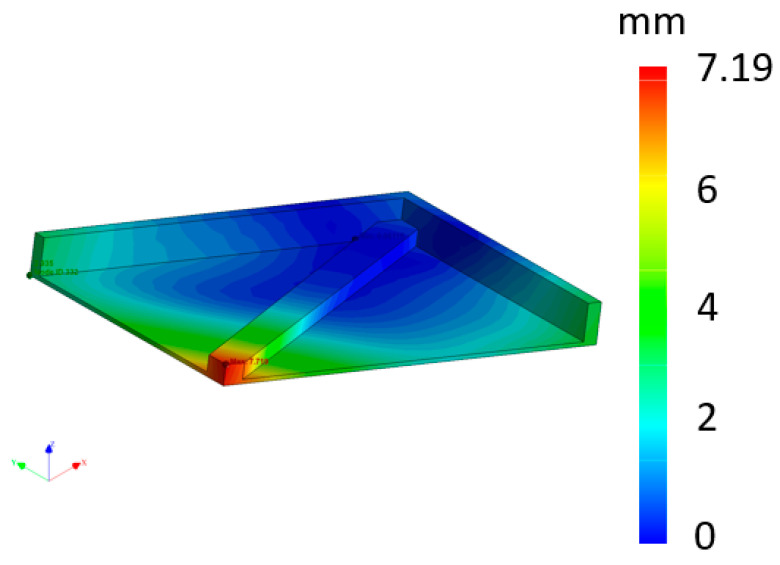
Simulated part in tilted position in the printer (mm).

The second simulation was of the piece tilted as it would be when printed in real life (approximately 45° orientation in the printing chamber). Here, the corner with the most material was at the lowest point (the corner where all three ribs were connected), meaning that it would be printed first. Figure 12 shows that the maximum deformation was located at the end of the rib, i.e., at the last printed area.

The deformation was caused by one main factor: the fact that the part had already begun to cool on the lower part, thus gradually creating greater deformation at the upper end of the part. The maximum deflection in this simulation was 7.72 mm. The difference between this and the previous result was caused by completely different heat transfer during the printing process.

**Figure 13:**
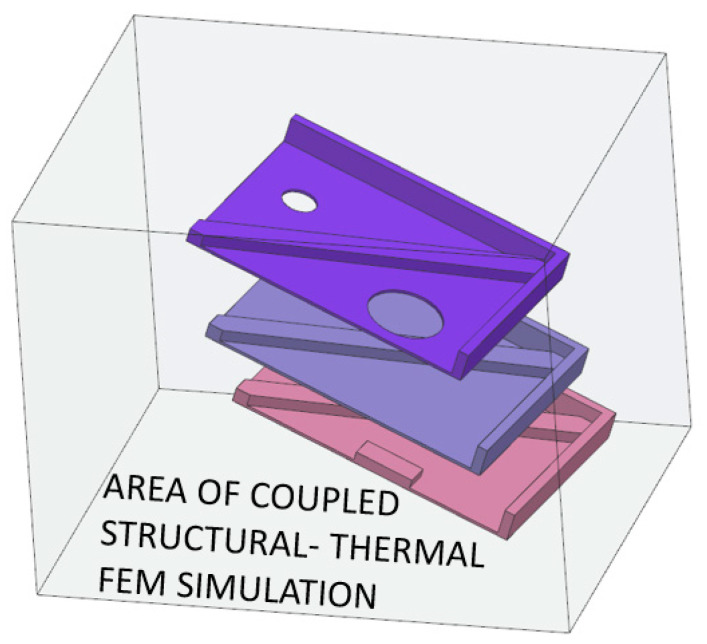
Positioning of parts for the simulation as in reality.

The last simulation was of the part at the same angle as before but this time as in the real world, with the part being printed at the same time as the two others (slightly different parts). Here, the simulation took place with three parts, as shown in Figure 13, but this research was focused only on the deformations developing on the initial part (the same part as in the previous simulations).

**Figure 14:**
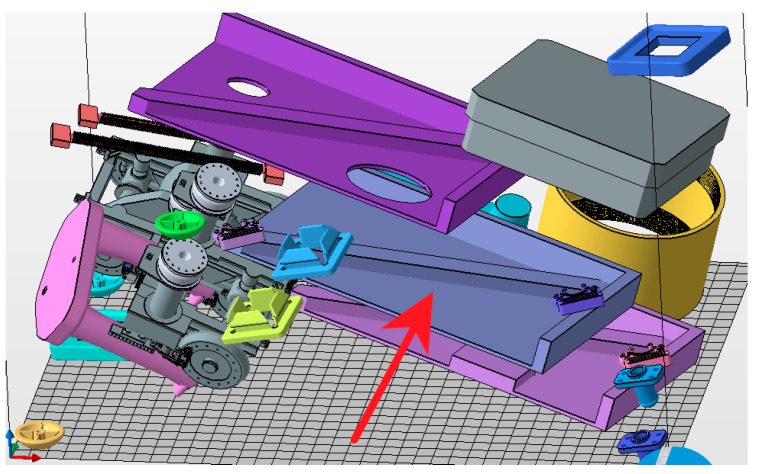
Parts in the HP MJF 4200 print box.

The part simulated in the previous section was printed as shown with the red arrow in Figure 14, which represents a different orientation than flat. It is obvious that the testing parts were produced within the print job with other parts. The thermal effect of these parts was neglected. These smaller parts were not part of this research; they were added to the print job by the printer operator, and the correct position of all parts was set up according to the official HP software Netfabb, which was used to communicate with the HP 4200 printer.

**Figure 15:**
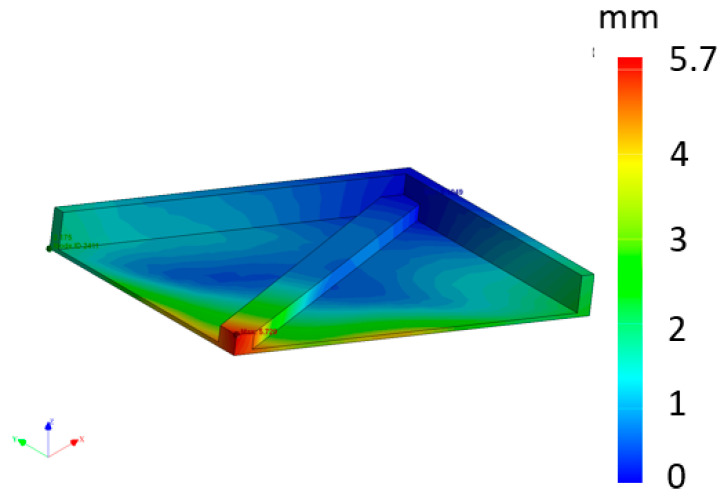
Simulated part with other parts (which are hidden) as in reality (mm).

The results were almost the same as those from the previous simulation; however, the deformations were less significant: 5.7 mm for this one (Figure 15) compared to 7.72 mm for the previous one. This is due to the fact that when a part is finished being printed, it remains warmer, and the printing of the other parts continues to heat the powder generally, thus increasing the cooling time of the part while homogenizing the temperature.

It can be concluded that the part deforms differently depending on the position in which it is printed. If minimal deformation is desired, it should be printed flat with other parts around it, even though it may tend to deform at multiple points. On the other hand, if a larger deformation at a single point is negligible, it might be preferable to print it inclined. Printing flat parts horizontally is generally minimally used because it causes problems with surface quality, such as sinks or ‘elephant skin’. This flat orientation is shown just for comparing. It will not be used in reality because it will cause the detailing and fusing agent to remain on the upper surface during printing.

## 5. Real Deflection

The measurement of deformations after the printing process has to be performed in order to validate the simulations from the virtual prediction. The difference between the simulation and reality has to be quantified.

**Figure 16:**
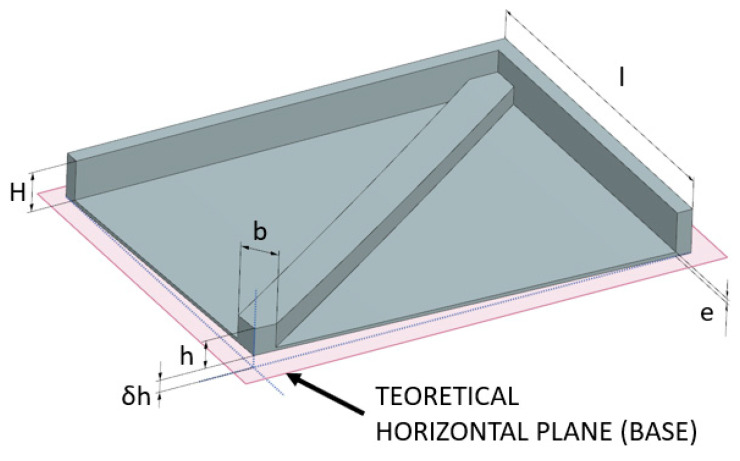
Printed part with measured parameters.

In order to check whether the simulated results are correct, it is necessary to observe the deformations that occur in the real part. Figure 16 shows the deformed printed part. The dimension points are shown in order to measure deflection in time.

### 5.1. Measurements

Measurements were taken at different times after the printing process. A total of 15 measurements (15 specimens) were performed at each time in order to obtain statistically correct results (by the 3D measuring station). The first measurement was just after the printing had finished, then two and five days later, and then every week for three weeks. To obtain long-term information, measurements were also taken six and eight weeks after printing.

From Table 3, it is clear that almost all deformations were negligible in view of the values achieved.

**Figure 17:**
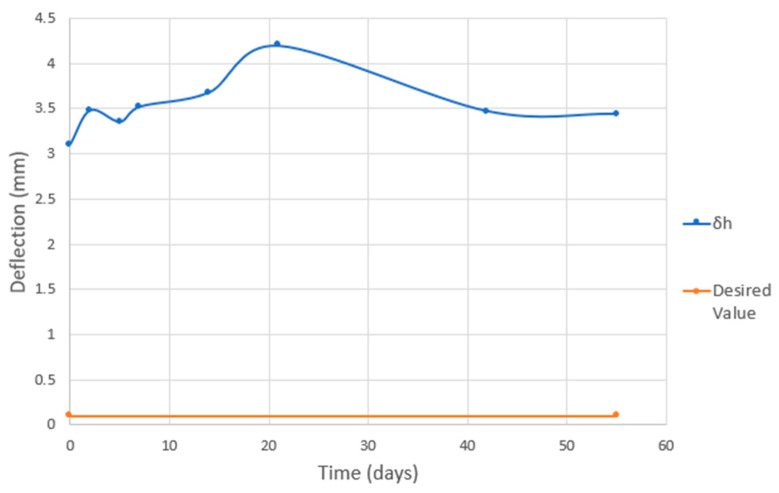
Measurement of the deflection at the end of the rib over time (δh).

The only non-negligible value was that at the end of the rib (δh), which should be zero. This value increased then decreased and seemed to stabilize at around 3.42 mm of deformation (Figure 17). It has to be mentioned that the part was designed specially without an opposite rib (nonbalanced design) in order to achieve some deformation to compare.

### 5.2. Discussion and Comparison of Results

By comparing the results of advanced simulations with the data obtained from real experiments, a correlation between the virtual world and the physical world can be established. It is obvious that the simulations and reality appeared to be consistent as the deformation occurred at the same point. However, there were a few differences between the values of the maximum deflection in the corner (δh).

When the part was simulated in a tilted position but alone (deformation: 7.19 mm), the value of the deformation was 1.49 mm greater than when the other parts were simulated at the same time (deformation: 5.7 mm). The difference between the simulation with the three parts together (deformation: 5.7 mm) and reality (deformation: 3.44 mm) was 2.26 mm. Finally, the difference between the simulated single part (deformation: 7.19 mm) and the real part (deformation: 3.44) was 3.75 mm. This is not an insignificant difference. Further research and development in this area must be carried out to bring virtual and real printing closer together and thereby design parts with minimal deformation. All results are shown in Table 4.

## 6. Conclusions

This study delved into the investigation of deformations that occur during the Hewlett-Packard (HP) Multi Jet Fusion (MJF) 3D printing process. The study focused on minimizing deformations by exploring different printing orientations and highlighting the factors that influence the deformation of printed parts.

Simulation results were compared with experimental data, demonstrating good consistency between the two, by considering the printing position, the material, the size, and even the quantity of pieces during printing.

The difference between reality (after stabilization of dimension in time) and simulation was 2.26 mm. This is the lowest achieved error with maximal accuracy of simulation for a full printing job. Further approximation of results is possible only by FEM mesh refinement. This is limited by the actual available computing power.

The insights gained into the deformation mechanisms and simulation methods provide valuable information for optimizing the design of parts printed using the HP MJF technology. This research contributes to enhancing the quality of printed parts and harnessing the full potential of the HP MJF technology across various industrial applications. In summary, this study underscores the significance of comprehending deformation mechanisms and employing effective strategies in order to minimize deformations during HP MJF 3D printing. Future research will continue with the description of material models of different composite polymers and refining results in order to minimize differences between simulations and the parts produced by the MJF (and other) technology. The main limit of this study is the limited computing power. The actual considered size of one element was 2 mm. This value should be lower than the layer size during the actual printing process (0.08 mm) in order to obtain more precise results. This means that elements that are 25 times smaller should be used for FEM simulations. This size of elements was not possible to simulate with the computational power that was used.

## Figures and Tables

**Table 1:** Engineering constants available from the Digimat MF library.

Parameters	PA12	GB	PA12GB
Density (g/cm^3^)	1.01	2.54	1.33
E (GPa)	1.735	72.000	2.685
G (GPa)	N/A	N/A	1.03
Ν	0.39	0.22	0.37
Yield stress	22	N/A	N/A
Hardening modulus	18	N/A	N/A
Hardening exponent	70	N/A	N/A
Linear hardening modulus	15	N/A	N/A

**Table 2:** Engineering constants from the manufacturer´s datasheet.

Parameters	PA12GB
Density (g/cm^3^)	1.30
E (GPa)	2.850
G (GPa)	1.03
Ν	0.21

**Table 3:** Deformations over time for the initial part in millimeters.

Value in Time (mm)	δh	h	H	l	e	b
Designed in CAD	0.00	10.00	20.00	150.00	2.00	16.45
Immediately after print	3.10	10.15	20.20	151.00	2.00	16.37
2 days after print	3.82	10.13	20.11	149.95	2.00	16.41
5 days after print	3.35	10.03	20.18	149.66	1.93	16.38
1 week after print	3.52	10.3	20.20	149.79	1.89	16.35
2 weeks after print	3.68	10.05	20.21	149.81	1.86	16.31
3 weeks after print	4.20	9.99	20.24	149.81	1.90	16.37
6 weeks after print	3.47	9.97	20.19	149.80	1.89	16.36
8 weeks after print	3.44	10.01	20.22	149.80	1.89	16.34

**Table 4:** Comparison of results.

	Desired Value in CAD	FEM—Flat Position	FEM—Tilted Position One Part	FEM—Tilted Position Three Parts	Steady State Value—Real Measurement
**δh**	0.00	1.03	7.19	5.70	3.44

## Data Availability

Data are contained within the article.
